# *KRAS* mutant allele-specific expression knockdown in pancreatic cancer model with systemically delivered bi-shRNA *KRAS* lipoplex

**DOI:** 10.1371/journal.pone.0193644

**Published:** 2018-05-31

**Authors:** Donald D. Rao, Xiuquan Luo, Zhaohui Wang, Christopher M. Jay, Francis C. Brunicardi, William Maltese, Luisa Manning, Neil Senzer, John Nemunaitis

**Affiliations:** 1 Strike Bio, Dallas, TX, United States of America; 2 University of Toledo College of Medicine and Life Sciences, Toledo, OH, United States of America; 3 Gradalis, Inc., Dallas, TX, United States of America; University of South Alabama Mitchell Cancer Institute, UNITED STATES

## Abstract

The *KRAS* oncogene, present in over 90% of pancreatic ductal adenocarcinomas, is most frequently the result of one of three gain-of-function substitution mutations of codon 12 glycine. Thus far, *RAS* mutations have been clinically refractory to both direct and selective inhibition by systemic therapeutics. This report presents the results of pre-clinical assessment of a lipoplex comprising a plasmid-encoded, modular bi-functional shRNA (bi-shRNA), which executes selective and multi-mutant allelic *KRAS*^*G12mut*^ gene silencing, encased within a fusogenic liposome systemic delivery vehicle. Using both a dual luciferase reporter system and a Restriction Fragment Length Polymorphism (RFLP) assay, selective discrimination of *KRAS*^*G12mut*^ from *KRAS*^*wt*^ was confirmed *in vitro* in PANC1 cells. Subsequently, systemic administration of the bi-shRNA^KRAS^ fusogenic lipoplex into female athymic Nu/Nu mice bearing PANC1 xenografts demonstrated intratumoral plasmid delivery, *KRAS*^*G12mut*^ knockdown, and inhibition of tumor growth, without adverse effect. Clinical trials with the bi-shRNA lipoplex have been implemented.

## Background

Pancreatic ductal adenocarcinoma (PDAC) is a disease characterized by early metastatic spread and high mortality. There has been limited benefit from the incremental changes in therapy of PDAC over the past 40 years despite an increased understanding of the genetic, epigenetic, biochemical and micro-environmental processes of this malignancy [1–4]. Mutations involving the proto-oncogene *KRAS* and tumor suppressors *CDKN2A*, *TP53* and *SMAD4* are the major genetic signal alterations responsible for malignant phenotype [2, 5, 6]. More than 90% of PDAC’s contain *KRAS* activating mutations, the majority of which are at codon G12 (COSMIC database). These mutated *RAS* family genes are key “pro-cancer” regulators of the RAF/MEK/ERK, PI3K/AKT/mTOR and RalA/B signaling pathways [4, 7]. Recently, *in vitro* and *in vivo* targeting of MEK, ERK, PI3K and mTOR in pancreatic cancer have shown promising results based on their ability to impede cellular growth or delay tumor formation. Several clinical trials have been initiated based on these results (NCI-2016-01356) [2, 8–10]. However, combinatorial toxicity and therapy-induced cross activation of collateral signaling pathways underscore the complexities of targeting the RAS downstream signaling pathway [3, 11, 12]. Murine models confirm the oncogenic driver status of *KRAS* mutation (*KRAS*^mut^) in PDAC in conjunction with the stepwise accumulation of additional genetic changes. Obstacles to effective direct targeting of *KRAS*^*mut*^ include a lack of well-defined binding pockets (“undruggability”), ineffective systemic delivery of RNA interference (RNAi) moieties and heretofore the lack of RNAi *KRAS*^*mut*^/*KRAS*^*wt*^ selectivity [1, 13, 14].

We report the results of pre-clinical assessment of a lipoplex comprising a plasmid encoded bi-functional shRNA (bi-shRNA) that executes selective *KRAS*^*mut*^ gene silencing encased within a fusogenic liposome systemic delivery vehicle. The unique mechanism of action of bi-shRNA has been described previously [15]. Briefly, the bi-shRNA-*KRAS*^*mut*^ consists of two stem-loop structures with a miR-17-92 backbone; the first stem-loop structure is composed of complementary guide and passenger strands, while the second stem-loop structure has strategic base pairing mismatches at key positions on the passenger strand. The encoding plasmid is able to accommodate mature shRNA loading onto more than one type of RNA-induced silencing complex (RISC) to effect both mRNA cleavage (via cleavage-dependent Ago2-loaded RISC) and mRNA degradation, *p*-body sequestration, and inhibition of translation (mediated by cleavage-independent Ago1-4-loaded RISC). Herein, using the PANC1 pancreatic cancer tumor model *in vitro* and *in vivo*, we demonstrate that *KRAS*^*mut*^ specific targeting with bi-shRNA-*KRAS*^*mut*^ effectively and selectively suppresses *KRAS*^*mut*^ expression and re-activates receptor tyrosine kinase (i.e., EGFR) signaling activity. This is the first demonstration of effective mutant-selective knockdown of *KRAS in vivo* with a systemically delivered therapeutic modality.

## Materials and methods

### Materials and cell cultures

HEK293, PANC-1 cells and ASPC-1 cells were purchased from ATCC (Manassas, VA). pSiCHECK vector was purchased from Promega (Madison, WI). HEK293 and PANC-1 cells were cultured in DMEM medium with 10% FBS, 2 mM Glutamine. ASPC-1 cells were cultured in RPMI-1640 medium with 10% FBS, 2 mM Glutamine. All cells were incubated in humidified incubator at 37°C with 5% CO2.

### Plasmids and construction

The bi-shRNAs were designed according to previously published protocols [16]. The bi-shRNAs expression units were constructed by gene-synthesis method, the synthetic DNA was sequence confirmed both before and after (Epoch Life Sciences, Missouri City, TX) uni-directional insertion into the Sal I and Not I sites of the pUMVC3 mammalian expression vector. Research grade plasmid DNA was prepared by Aldevron (Fargo, ND).

### Cell transfection

Cells were transfected either by electroporation (Gene Pulser II Electroporation System; Bio Rad) or by reverse transfection method using Lipofectamine 2000 reagent (Invitrogen).

### Dual luciferase assays

Dual luciferase activity was assayed in a 96-well plate with Dual-Luciferase® Reporter Assay System purchased from Promega (Madison, WI). The activities of firefly and *Renilla* luciferases were measured sequentially from a single sample. The firefly luciferase reporter was measured first; after quantifying the firefly luminescence, this reaction was quenched, and the *Renilla* luciferase reaction was simultaneously initiated by adding Stop & Glo® Reagent to the same well. HEK293 cells were transfected by electroporation. Afterwards, cells were plated in 96-well plates in triplicate and assayed at 24, 48 or 72 hours post transfection. The fluorescence was read by Gemini XPS Microplate Reader (Molecular Device).

### Cell viability assay

Cell viability was assayed using the CellTiter-Blue® Luminescence Cell Viability Assay System from Promega (Madison, WI). Transfected cells or treated cells were plated in triplicate in 96-well plates and assayed at 24, 48 or 72 hours post transfection. Cells were lysed and assayed with reagents supplied by the assay system and the fluorescence was detected by using Luminoskan™ Ascent Microplate Luminometer (ThermoFisher Scientific).

### DNA and DNA-Lipoplex

50 mg of research grade plasmid DNA was contract manufactured by Aldevron (Fargo, ND). The identity of manufactured plasmids was reconfirmed by restriction digest and by sequencing the insert region before DNA-lipoplex manufacturing. The lyophilized DNA-Lipoplex was manufactured according to the thin film Liposome method as previously published (Templeton, Nature Biotech 1997 and Phadke, DNA and Cell Biol 2011) with the following modifications: after the rotovap step to create the DOTAP:Cholesterol film, the product was resuspended in 10% sucrose and then manually extruded through successively smaller pore size filters to create the Liposomes. The Liposomes were mixed with DNA to create the DNA-Lipoplex product and intermediate QC was performed to check specifications. The product was vialed, frozen, and lyophilized overnight. The following day, the freeze-dried product was sealed, labeled, and quarantined for QC/release.

After the product was released and ready for use, the freeze-dried DNA-Lipoplex was reconstituted in 5% dextrose and extruded through a 1.0 μm filter. At this point, the product was ready for use and could be directly injected or diluted with additional 5% dextrose to the appropriate concentration prior to administration.

### Ethanol injection DNA-Lipoplex production

The aqueous DNA-Lipoplex was manufactured according to the two-step ethanol injection method previously published (Rao, Mol Therapy 2016).

### Restriction fragment length polymorphism (RFLP) assay

The RFLP method is schematically presented in S1 Fig. Specifically, total cell RNA was isolated and treated with DNase using RNeasy Mini (Qiagen). 2–10 μg of total RNA was reverse transcribed with *KRAS* gene-specific primer Kras 001 (CTTGCTTCCTGTAGGAATCCTCT) in a 20 μl reverse transcriptase reaction using IScript SelectKit (Bio-Rad) and a Bio Rad thermal cycler. Fraction of cDNA was amplified by polymerase chain reaction (PCR) using primer set Kras 014 (ACTGAATATAAACTTGTGGT***CCA***TGGAGCbxT) and Kras 001 (CTTGCTTCCTGTAGGAATCCTCT). The PCR amplicon is 127bp. Primer Kras 014 introduces a Bst XI site, which recognizes Kras WT sequence and cuts the PCR amplicon to 99 bp and 28 bp fragments, Bst XI does not recognize mutant G12D sequence. Both digested (Bst XI) and undigested PCR products were electrophoresed on a 4% agarose gel to score WT transcripts and mutant transcripts. In addition, the digested PCR amplicons were analyzed on Experion (Bio-Rad) with improved detection sensitivity. 1μl of digested amplicons was loaded onto a DNA 1K chip and the fragments were visualized and analyzed by the Experion analysis software.

### Plasmid detection and quantification

The mouse tissues were thoroughly homogenized using Qiagen TissueLyzer II. Tissue homogenate was then digested with proteinase K and the total DNA extracted using DNeasy Kit (Qiagen). The plasmid was detected and quantified using a home-developed qPCR assay. Briefly, 2ul of extracted total DNA was mixed with a BioRad IQ Supermix, a pUMVC3 forward primer, a pUMVC3 reverse prime and a TaqMan probe specifically recognizing the pUMVC3 amplicon. The 40 cycle qPCR program (using a BioRad CFX384 qPCR instrument) was set up using an automatic liquid handler. The DNA copy number was quantified by referring the Ct numbers to a standard curve.

### *In vivo* mouse xenograft study

This study was carried out in strict accordance with the recommendations in the Guide for the Care and Use of Laboratory Animals of the National Institutes of Health. The protocol was approved by the Committee on the Ethics of Animal Experiments of the Altogen Labs, Austin, TX (IACUC protocol 3–17836). Following modifications were made to the study “endpoint” definition: Moribund animals or tumor xenograft volumes of 2,000 mm^3^, or 40 days after xenotransplantation. All surgery was performed under sodium pentobarbital anesthesia, and all efforts were made to minimize suffering. Immune-compromised nude mice (9- to 11-week old females) were purchased from the Harlan laboratories. All animal procedures and maintenance were conducted in accordance with the institutional guidelines (Altogen Labs, Austin, TX). The maximum tumor size was 2,000 mm^3^. Animals were observed at 6–8 hours and 1 day after each injection for acute reaction; neither adverse reactions nor aberrant behavioral phenotypes were observed.

### Observation and data collection

After tumor cells inoculation, the animals are checked daily for morbidity and mortality. At the time of routine monitoring, the animals are checked for any adverse effects of tumor growth and treatments on normal behavior such as mobility, visual estimation of food and water consumption, body weight gain/loss, eye/hair matting, pain/distress, self mutilation, and any other abnormal effects. Signs of graft rejection, infection, and unalleviated pain will be justification for immediate euthanasia as determined by the veterinarian.

Tumor volumes are measured every 3–4 days in two dimensions using an electronic caliper, and the volume data are expressed in mm^3^ using the formula: V = 0.5 *a* x *b*^2^ where *a* and *b* are the long and short diameters of the tumor, respectively. Dosing and tumor volume measurement procedures are conducted in a Laminar Flow Cabinet according to Altogen Labs IACUC regulations.

### Group assignment

Before grouping and treatment, all animals are weighed and the tumor volumes confirmed (100-150mm^3^) using electronic caliper. Since the tumor volume can affect the effectiveness of any given treatment, mice assigned into groups using randomized block design as following: First, the experimental animals are divided into homogeneous blocks based on their tumor volume. Secondly, within each block, randomization of experimental animals to different groups conducted. By using randomized block design to assign experimental animals, we ensure that each animal has the same probability of being assigned to any given treatment groups and therefore systematic error is minimized.

### Clinical observations

**Fig 1:**
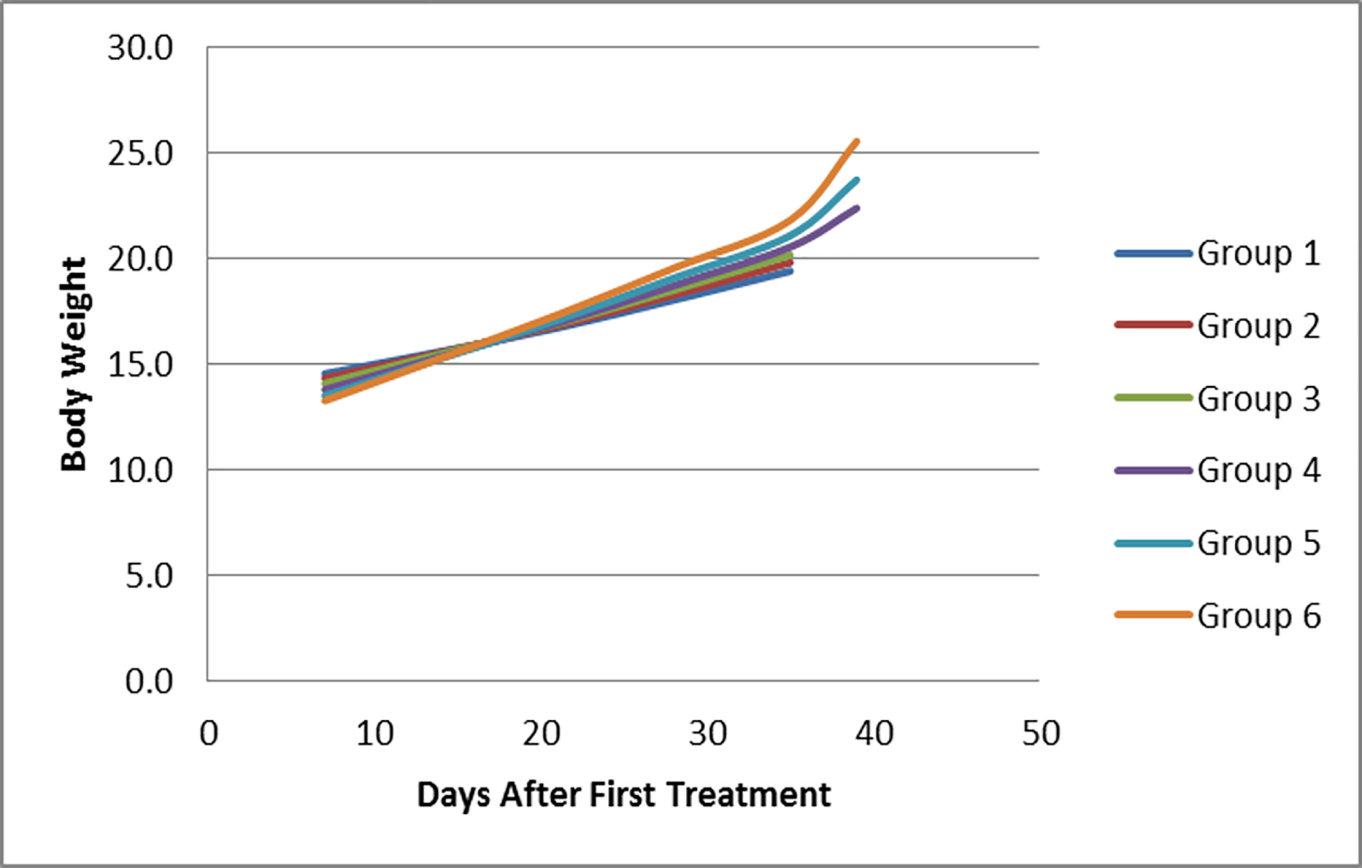
*In vivo* treatment show no adverse effect with PANC-1 xenograft model. Average body weight of animal with the same grouping as Fig 4A.

There was no clinical signs or behavioral phenotype observed within the study (daily cage intensive observation for adverse effect were performed). No BWL>20% were observed in any of the groups (Fig 1).

Tumor size was measured every 4 days. Animal body weight (g) was measured in subcutaneous PANC1 xenografts on days 7, 14, 21, 28, 35, 39 after tumor inoculation (Day 0); no significant changes were observed. At the end of study, animals were sacrificed by cervical dislocation. The pancreas carcinoma PANC1 (CRL-1469) cell line was obtained from ATCC and cultured in ATCC formulate Dulbecco's Modified Eagle's Medium (cat#302002) and supplemented with fetal bovine serum to a final concentration of 10% (ATCC). Subculturing was performed by trypsinization with 0.25% Trypsin-EDTA (2–3 minutes) and 1:4 split for every subsequent passage. The cell line was cultured at 37°C / 5% CO_2_ in a humidified incubator. Cells were mixed (1:1 volume) with Matrigel (BD Biosciences) and the suspension (50% matrigel) subcutaneously injected (1.0 x 10^6^ cells per injection) on day 0 into the animal flank area to ensure successful tumor initiation and tumor growth measurements. Ninety animals were used for PANC-1 xenotransplantation and 78 animals with measurable tumors were selected on day 7 to be used for subsequent experiments. 10 animals per group (n = 6) were used for growth inhibition study and 3 animals per group (n = 6) were used for molecular analysis.

Freeze-dried formulations used for this study were reconstituted immediately prior to each injection. Reconstitution was performed in a Biological Safety Cabinet. A 3 mL syringe and 16 G needle was used to transfer D5W into the vial containing the freeze-dried test article, which was then gently flicked to resuspend the test article (final concentration = 0.25 mg DNA/ml) and then finally filtered with a 1.0 μm PES syringe filter. The filtered test article was pooled and diluted with D5W to the final injection dose (200μl per animal).

The compound or control was Intravenously administered on day 7 post-inoculation when measurable tumor growth was detected with an average tumor size of 150 mm^3^. Study mice were randomly assigned to each study group with an equal distribution of tumor size per group. Each group comprised 13 animals. 10 of the animals were enrolled in tumor growth inhibition study. Measurements of tumor volume (mm^3^) were performed by digital calipers every 4 days for 40 days post tumor inoculation. Animal body weight (g) was measured in subcutaneous PANC1 xenografts on days 7, 14, 21, 28, 35, 39 after tumor inoculation (Day 0). Remaining 3 animals in each group were sacrificed at day 27 (two days post last infusion) from which tumors were harvested for molecular analysis.

### Western blot

To prepare total protein lysates, half of the PANC1 tumor tissue samples preserved in Allprotect Tissue Reagent (Qiagen) were first cut out and weighted, then ice cold CelLytic MT^TM^ Cell Lysis Reagent for mammalian tissues (Sigma, St. Louis, MO) with 1 x protease inhibitors (Sigma), 1 x phosphatase inhibitors 2 (Sigma) and 1 x phosphatase inhibitors 3 (Sigma) was added at a ratio of 10 μL lysis buffer per 1 mg tumor sample. Tumor tissue was homogenized and incubated on ice for 30 minutes. The homogenates were centrifuged at 14,000 rpm for 10 min and the supernatants transferred to a clean tube. The protein concentration was measured using the Bradford method. 30 μg total protein lysate of each tumor sample was mixed with 1/3 volume of 4 x Laemmli Sample buffer (Bio-Rad, Hercules, CA) with 5% β-mercaptoethanol (Sigma) and denatured by heating, then loaded on 4–20% Mini-PROTEAN TGX gradient gel (Bio-Rad). After electrophoresis, the protein was transferred to a PVDF membrane using Trans-Blot Turbo system (Bio-Rad). To detect target protein, the membrane was first blocked with 5% Blotting-Grade Blocker (Bio-Rad), 0.1% Tween 20 (Sigma) in 1 × DPBS for 1 hour at room temperature on an orbital shaker, then probed with primary antibody, 1:1000 dilution, at 4°C overnight in 5% Blotting-Grade Blocker (Bio-Rad), 0.1% Tween 20 in 1 × DPBS on orbital shaker. After washing the membrane with 0.1% Tween 20 in 1 × DPBS 3 times, 5 minutes each, the membrane was probed with HRP conjugated anti-mouse or rabbit 2^nd^ antibody (Santa Cruz) at 1:2000 dilution in 5% Blotting-Grade Blocker (Bio-Rad), 0.1% Tween 20 in 1 × DPBS for 2 hours on the orbital shaker. Then the membrane was washed three times with 0.1% Tween 20 in 1 × DPBS 3 times, 5 minutes each. The signal was detected using SuperSignal West Dura Chemiluminescent Substrate (Thermo Scientific) and captured using G-Box (Syngene). For phosphorylated proteins, primary antibody targeting to specific phosphorylated site was first probed, then the membrane was striped with Restore Western Blot Stripping Buffer (Thermo Scientific) for 10–15 minutes at room temperature. The effect of stripping was verified by confirming no residual signal using G-Box with SuperSignal West Dura Chemiluminescent Substrate for 15–30 minutes. Then the same membrane was probed with primary antibody targeting the total protein to visualize the amount of total protein. The densitometry of the protein signal was calculated using software Image J. The primary antibodies: anti-EGFR (D38B1), anti-phospho-EGFR (Y1068) (D7A5), anti-Akt (11E7), anti-phospho-Akt (S437) (D9E), anti-phospho-Akt (T308) (244F9), anti-p44/42 MAPK (Erk1/2) (137F5), anti-phospho-p44/42 MAPK (Erk1/2) (197G2), anti-MEK1/2 (47E6), anti-phospho-MEK1/2 (Ser217/221) (41G9) and anti-Ras (D2C1) were obtained from Cell Signaling (Boston, MA). Anti-phospho-EGFR (Y1125) was obtained from WuXi AppTec (San Diego, CA), anti-phospho-EGFR (Y1069, referred as Y1045 in this paper) from Upstate (Lake Placid, NY), and anti-GAPDH from Santa Cruz (Dallas, TX).

## Results

### Use of a dual luciferase reporter system to optimize *KRAS*^*mut*^ mutant specific knockdown constructs

**Fig 2:**
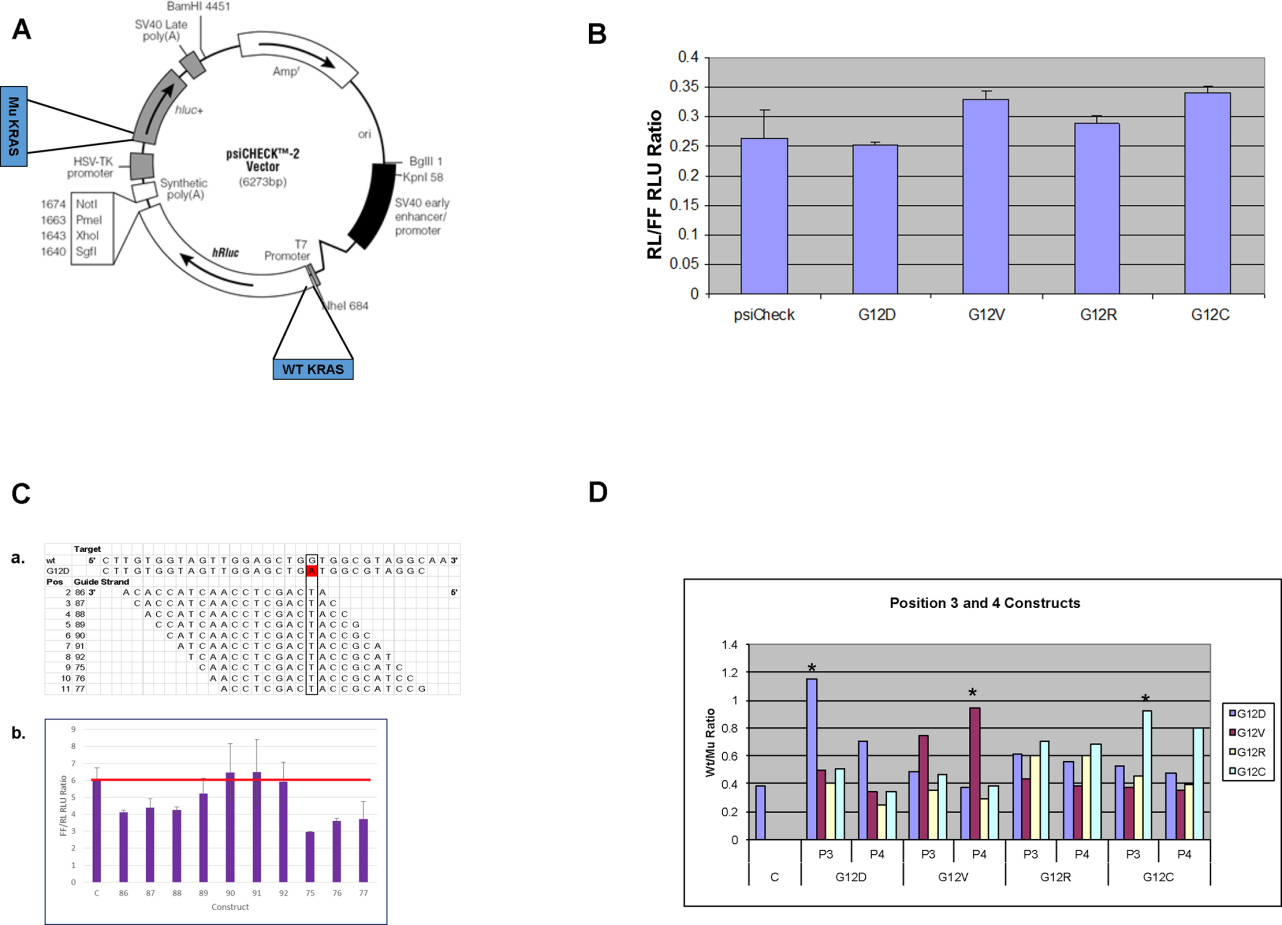
Dual luciferase system to identify the most optimum mutant selective constructs. Coding sequences for the first 17 amino acids of *KRAS* wild-type (wt) and mutant (mu) were inserted into the amino terminus of the psiCHECK2’s hRluc (renilla) and hluc (firefly) coding sequence, respectively. Knockdown of wt vs. mu sequence is compared by renilla to firefly intensity ratio. **A.** Schematic of the sequence insertion into psiCHECK2 for reporter constructs. **B.** Bar graph show comparison of reporter constructs relative light unit (RLU) intensity ratio of renilla (RL) to firefly (FF). Y-axis is RL/FF RLU ratio. X-axis is reporter test vectors and parent reporter vector. Standard deviation bar represents measurement from quadruplet samples of independently transfected cells in 96-well format and assayed simultaneously post transfection. **C.** Positional effect of G12D knockdown constructs; panel **a**: Table illustrate each constructs guide strand sequence in relation to G12D mutation site. 1^st^ column indicates position of G12D mutation in guide strand of each construct. 2^nd^ column is the code for each construct. The guide strand sequence is shown as the complement of target sequence at 3’ to 5’ orientation; panel **b**: Bar graph show comparative plot of FF/RL RLU ratio (mu/wt) for each knockdown construct. Sample C is the control without knockdown vector. The red bar represents average control sample value for visual enhancement. Standard deviation bar represents measurement from quadruplet samples of independently transfected cells in 96-well format and assayed simultaneously post transfection. Two-tailed student T-test indicates ρ-value ≤ 0.05 between control and samples 86, 87, 88, 75, 76 and 77. **D.** G12D, G12V, G12R and G12C knockdown constructs (with mutation nucleotide at position 3 or 4 of the guide strand) were tested against test reporter vectors of all four mutations. Bar graph shows the summary of relative average FF/RL RLU ratio (mu/wt). * indicate the most effective constructs for each mutation. X-axis is the knockdown constructs for G12D, G12V, G12R and G12C. P3 indicate knockdown construct with mutated nucleotide at position 3 of the guide strand. P4 indicate knockdown construct with mutated nucleotide at position 4 of the guide strand. Y-axis is the FF/RL RLU ratio.

The psiCHECK2 reporter vector is a mammalian expression vector that expresses dual luciferase reporters on a single vector thereby allowing testing of two expressed sequences in the same environment under the same conditions. We inserted nucleotide sequences encoding the first 17 amino acids of *KRAS* into the regions encoding the amino terminal of the psiCHECK2 vector luciferase reporter genes; i.e., the *KRAS*^*wt*^ sequence was inserted into the renilla (RL) luciferase gene and a *KRAS*^*mut*^ sequence was inserted into the firefly (FF) gene (see Fig 2A; for specific sequence insertion see S2A Fig). A total of five psiCHECK2- based test vectors were constructed: G12D, G12C, G12V, G12R, and one for the wild-type sequence only (S2A Fig). The test reporter constructs express a RL/FF ratio similar to the parent psiCHECK2 (Fig 2B). A single nucleotide G→A change leads to G12D mutation. Using tiling approach, we constructed a series of bi-shRNA knockdown vectors with the G12D mutation’s single nucleotide change positioned at positions 2–11 of the guide strand (Fig 2C, panel a). We then co-transfected the G12D/WT dual expression test vector with the G12D specific knockdown vector and showed that positioning of the mutant sequence at different positions of the guide strand resulted in different FF/RL ratios (Fig 2C, panel b). Slight variations in the test vector to knockdown vector ratio showed similar reproducible results (S2B Fig). All subsequent studies were done with the test vector to knockdown vector at 1 to 1 ratio. An advantageous knocked down mutant:wild-type ratio was obtained with complement to mutated nucleotide in positions 2, 3, 4, 5, 9, 10 and 11 of the guide strand. Complement to mutated nucleotide at positions 2, 3 or 4 of the guide strand were the most effective vis-a-vis selective mutant sequence knockdown not having a significant effect on wild-type transcripts, whereas substitutions at positions 7, 8, 9 and 11 reduced wild-type expression as well (S2C Fig). Position 3 and 4 substitutions for G12V, G12R and G12C were constructed and similarly tested; the comparative results are shown in Fig 2D. The results of the screening effort are summarized in S1 Table.

### Triplex bi-shRNA-*KRAS*^*mut*^ constructs can effectively and specifically knockdown *KRAS*^*mut*^ expression without affecting *KRAS*^*wt*^ expression in cultured cells

**Fig 3:**
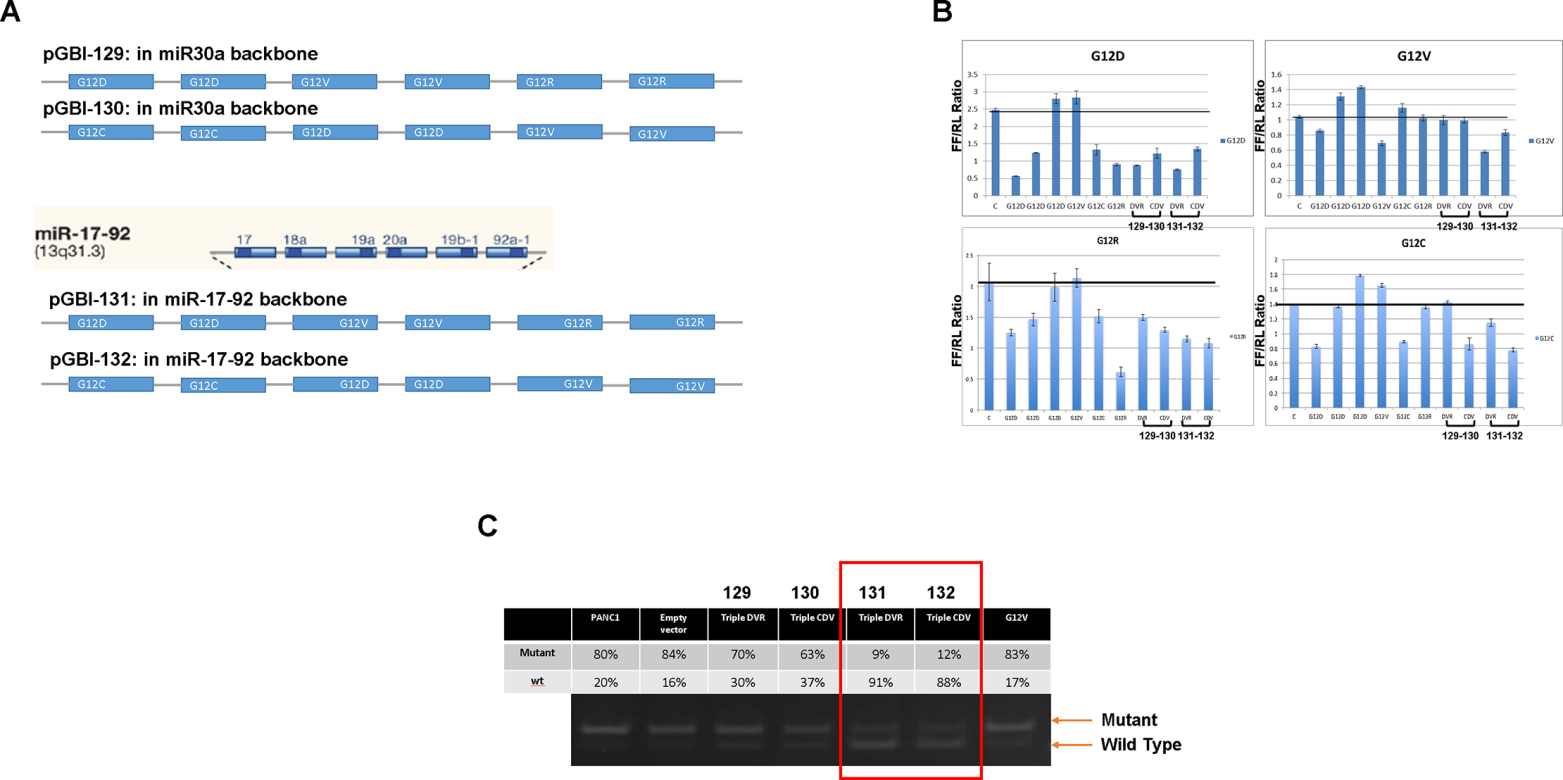
Triple mutant knockdown constructs and mutant allele selective knockdown. **A**. Schematics shown expression unit sequence arrangement of triple knockdown constructs in either miR30a backbones or miR-17-92 cluster backbones. **B.** Bar graphs show analysis of the specificity of triple knockdown constructs by reporter vectors. For each bar graph, the Y-axis is FF/RL RLI ratio (mu/wt) and the X-axis is knockdown vectors tested. At the top of each panel indicate individual mutant targeting vectors tested. Samples from left to right: C = control, G12D = G12D knockdown vector (three independent knockdown vectors with different efficiencies; G12D specific knockdown vector with mutated nucleotide at position 2, position 3 and position 6 of the guide strand, respectively), G12V = G12V knockdown vector with mutated nucleotide at position 4 of the guide strand, G12C = G12C knockdown vector with mutated nucleotide at position 3 of the guide strand, G12R = G12R knockdown vector with mutated nucleotide at position 4 of the guide strand, DVR = triple knockdown vectors for G12D, G12V and G12R in miR30a backbone (code named 129), CDV = triple knockdown vectors for G12C, G12D and G12V in miR30a backbone (code named 130), DVR = triple knockdown vectors for G12D, G12V and G12R in miR17-92 backbone (code named 131), CDV = triple knockdown vectors for G12C, G12D and G12V in miR17-92 backbone (code named 132). The mutated nucleotide position at the guide strand of the triple constructs for G12D, G12V, G12C and G12R were guide strand position 3, 4, 3 and 4, respectively. Standard deviation bar represents measurement from quadruplet samples of independently transfected cells in 96-well format and assayed simultaneously post-transfection. **C.** Elecropherogram show RFLP of *KRAS* mRNA in PANC-1 cells stably transformed with triple knockdown vectors. Each individual lane is labeled with their respective % of mutant vs. wild *KRAS* transcripts. % of mutant vs. wild-type was assessed by electrophaerogram band intensity scan. Samples PANC1 = non-transformed parent PANC-1 cells, Empty vector = non-transformed parent PANC-1 cells transfected with empty vector (pUMVC3 with no insert), Triple DVR (129) = PANC-1 cells transformed with DVR triple knockdown vector in miR-30a backbone (code named 129), Triple CDV (130) = PANC-1 cells transformed with CDV triple knockdown vector in miR-30a backbone (code named 130), Triple DVR (131) = PANC-1 cells transformed with DVR triple knockdown vector in miR-17-92 backbone (code named 131), Triple CDV (132) = PANC-1 cells transformed with CDV triple knockdown vector in miR-17-92 backbone (code named 132), G12V = PANC-1 cells transformed with G12V knockdown vector.

Given that the majority of oncogenic *KRAS* mutations are at codons 12 and 13, we designed a single transcription unit capable of a broadened range of *KRAS* mutant knockdown. Two sets of triplex knockdown vectors were constructed; one for G12D, G12V and G12R (51%, 30% and 12% of PDAC *KRAS* mutations, respectively) and another one for G12C, G12D and G12V (prevalent in colorectal and lung adenocarcinoma [17]). The most effective and discriminating of the G12D, G12V, G12C and G12R knockdown bi-shRNA cassettes were included in the triplex constructs; the guide strand location of each mutated nucleotide at positions 3, 4, 3 and 4, respectively. We also evaluated the polycistronic miR-17-92 cluster backbone [designated constructs 131 (bi-shRNA^DVR^) and 132 (bi-shRNA^CDV^)] as an alternative to the miR-30a backbone [constructs 129 (bi-shRNA^DVR^) and 130 (bi-shRNA^CDV^)] (schematically shown in Fig 3A; the sequences in S3A and S3B Fig). Using the dual reporter system, we demonstrated that all triplex constructs produced selective G12D, G12V, G12C and G12R knockdown albeit with varied efficiency (Fig 3B). For G12D and G12R, all four constructs were effective, although the latter was less so than the G12R specific bi-shRNA construct. Construct 131 was most effective for G12V and constructs 130, 131 and 132 for G12C. Based on effectiveness, miR-17-92 was chosen as the generic backbone for multiplex constructs.

*KRAS* G12D heterozygous PANC1 cells were co-transfected with both knockdown and neomycin resistance expression vectors and then selected for G418-resistant stably transformed cells for Restriction Fragment Length Polymorphism (RFLP) assay to discriminate *KRAS*^*mut*^ from *KRAS*^*wt*^ (see S3C Fig). Non-transformed cells, empty vector cells and non-specific G12V knockdown vector transformed cells all showed mutant transcript comprising 80–84% of total *KRAS* transcripts (Fig 3C, lanes 1, 2 and 7). There was proportionally less mutant transcript (63–70%) with constructs 129 and 130 (Fig 3C, lanes 3 and 4), whereas constructs 131 and 132 reduced the mutant transcript proportion to 9–12% of the total or 10.7–14.3% of that seen with the empty vector mutant (Fig 3C, lanes 5 and 6). Interestingly, the total amount of *KRAS* transcript (mutant + wild-type) was the same in control and knockdown cells.

### Position specific bi-shRNA-KRAS^mut^ constructs reduce PANC1 cell growth *in vitro*

Pancreas ductal adenocarcinoma cell PANC1 with *KRAS*^*G12D/wt*^, human embryonic kidney cell HEK293 with *KRAS*^*wt/wt*^ and colorectal cancer cell HT29 with KRAS^wt/wt^ were tested for growth inhibition *in vitro*. In so far as high doses non-discriminatively inhibited cell growth presumably due to non-specific transfection effect, we determined 10 ng per 96 well as the optimum dose and used that in this series of studies. Mutant nucleotide placement at positions 2, 3, 4, 7, 8, and 9 of the guide strand effectively inhibited PANC1 cell growth after 24 hours post transfection (S4A Fig). Growth of HEK293 cells was somewhat affected with mutant nucleotide at position 7, 8, 9, 10 of the guide strand (S4B Fig). No growth effect was observed for HT29 cells.

### Triplex bi-shRNA*-KRAS*^*mut*^ constructs effectively reduce tumor xenograft growth *in vivo*

**Fig 4:**
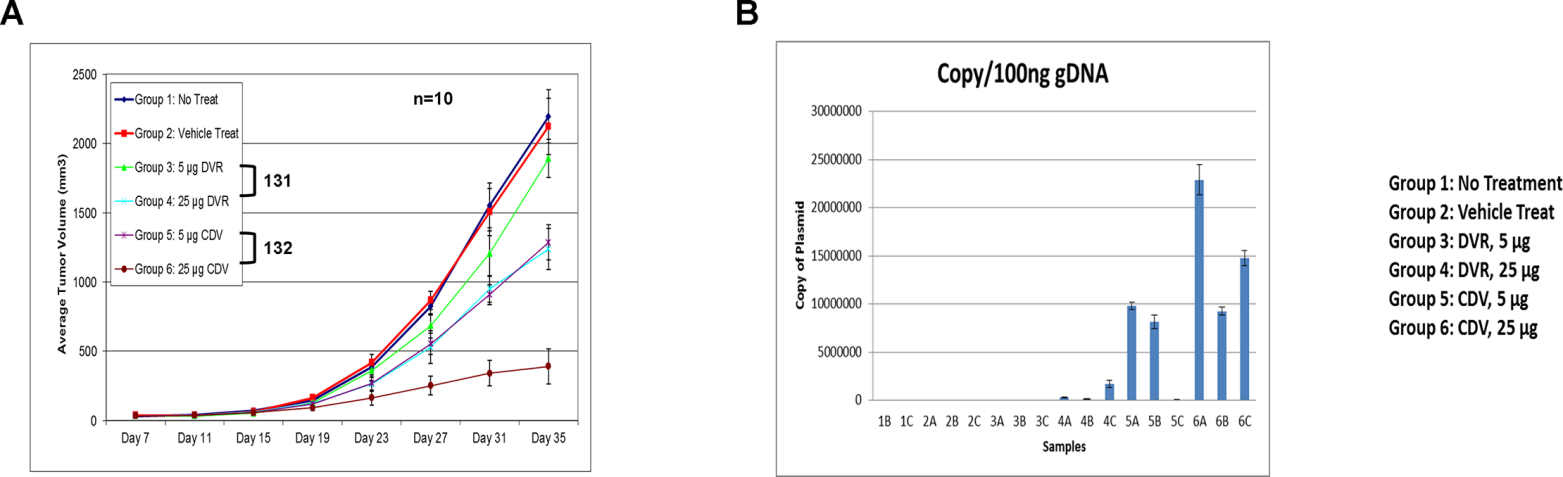
PANC-1 xenograft model. **A.** Average tumor volume measurement of PANC-1 tumor xenograft. Group 1: no treatment (blue line), Group 2: vehicle treated (red line), Group 3: 5 μg of 131 (DVR triple knockdown in miR17-92 backbone, green line), Group 4: 25 μg of 131 (DVR triple knockdown in miR17-92 backbone, light blue line), Group 5: 5 μg of 132 (CDV triple knockdown in miR17-92 backbone, purple line), Group 6: 25 μg of 132 (CDV triple knockdown in miR17-92 backbone, dark red line). **B.** Bar graph show average copy number of plasmids per 100 ng of genomic DNA found in tumor samples. The same treatment grouping as for panel A, samples A, B, or C represents three different tumors from three different animals of the same treatment group.

1x10^6^ PANC1 cells were subcutaneously implanted in female athymic Nu/Nu mice and treatment started when tumor volume reached 150 mm^3^. Based on *in vitro* activity (Fig 3C), 5 μg or 25 μg per infusion of the bi-shRNA^KRAS^ fusogenic lipoplex constructs 131 or 132 were administered via slow tail vein injection twice weekly for four weeks. The lipoplex formulation was freeze-dried and stored at 4°C, then reconstituted and filtered prior to each application. Tumor growth was inhibited by both constructs in a dose-dependent manner, with 132 being the most effective (Fig 4A). Treatments were well tolerated and weight loss was not observed. Tumor sampling confirmed intratumoral plasmid delivery and copy number correlation with administered dose (Fig 4B). We postulate that the discrepancy in delivery and consequent decreased growth inhibition of construct 131 resulted from the re-constitution and filtration process of the freeze-dried formulation (Fig 4A, groups 3 and 4).

### Treated tumor samples show *KRAS* mutant specific knockdown and activation of EGFR signaling *in vivo*

**Fig 5:**
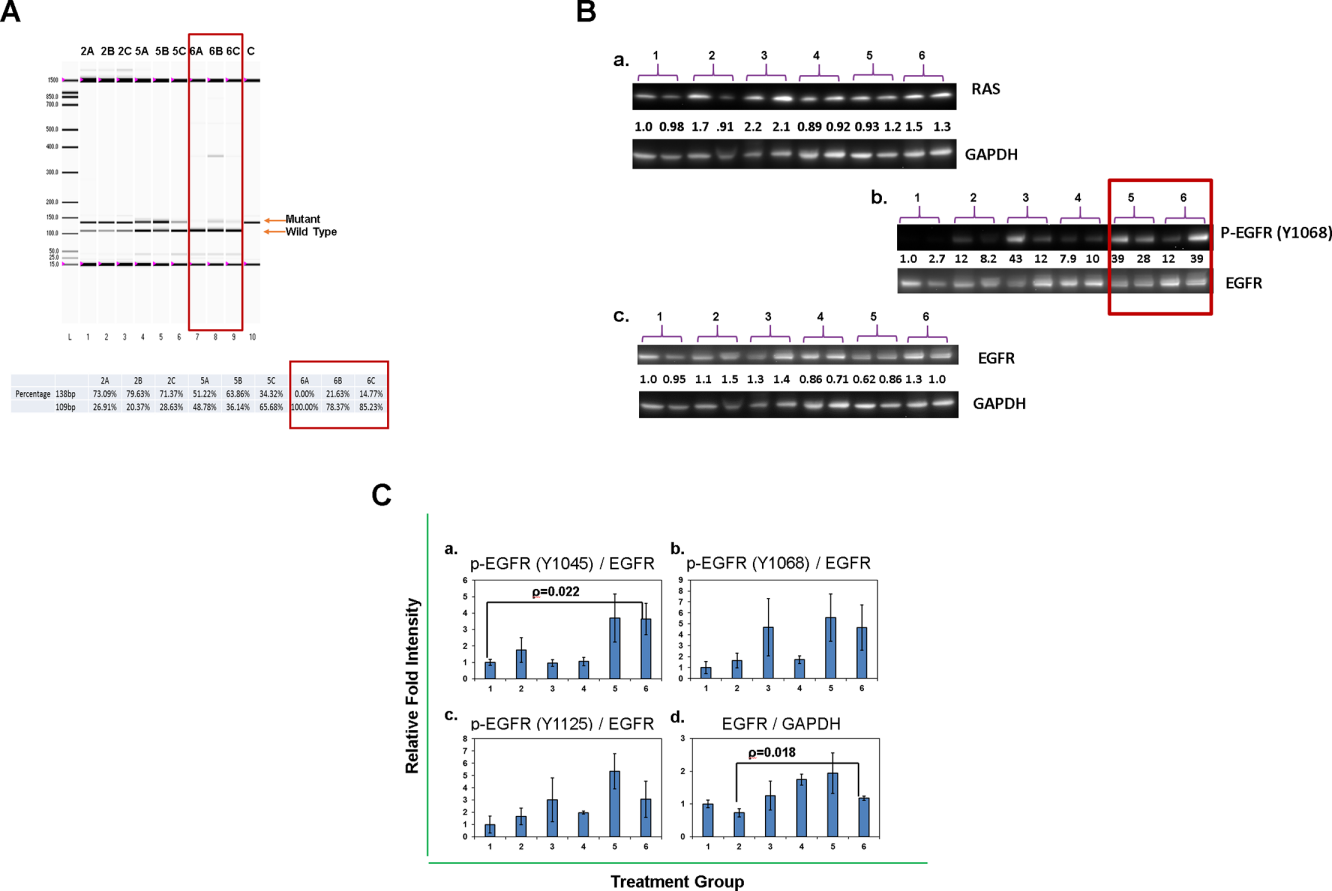
Molecular analysis of *in vivo* tumor samples. **A.** Electropherogram analyze % of mu and wt *KRAS* transcripts in *in vivo* treated tumor samples. Tumors were removed from animals after four weeks of various treatments, proportions of mu and wt *KRAS* transcripts were analyzed by RFLP and assayed by Experion. Sample designation is the same as indicated for Fig 4B. % mu and wt *KRAS* transcripts show at the bottom of the figure was determined by Experion software. **B.** Western transfer shows protein expression in various tumor samples. Numbers on each sample indicate treatment groups as presented in Fig 4. Two independent isolated tumors are analyzed for each treatment group. Panel a shows RAS protein in various treated tumor samples normalized against GAPDH. Panel b shows p-EGFR at position Y1068 quantitatively normalized against total EGFR protein. Panel c shows total EGFR protein for various treatment groups. **C.** Bar graphs summarize fold intensity difference from various groups of *in vivo* samples. Sample groupings are the same as shown on Fig 4. Panel a is for p-EGFR at Y1045 normalized to total EGFR protein. Panel b is for p-EGFR at Y1068 normalized to total EGFR protein. Panel c is for p-EGFR at Y1125 normalized to total EGFR protein. Panel d is total EGFR protein normalized to GAPDH. For each sample n = 3. Bar graphs shown are data obtained from approximately half of tumor of three independent animals. Standard deviation bar represents measurements of tumor from three animals. With one tailed, equal variances, student T-test, the following samples show statistical significant ρ-value ≤ 0.05: Panel a between samples 1 and 6, Panel c between samples 1 and 5, Panel d between samples 1 and 4, 2 and 4, 2 and 6.

Tumors were sampled from each treatment group at two days after the sixth treatment. Tumors were preserved with Qiagen AllProtect after harvesting and subsequently stored at -20° C before processing for molecular analysis. One half of each preserved tumor was analyzed by RFLP to determine the wild-type to mutant *KRAS* mRNA transcript ratio. Empty liposome treated group 2 and construct 132 lipoplex (S3B Fig) treated groups 5 and 6, were examined and compared (Fig 5A). The WT/mutant transcript ratio of the empty liposome control treated group was approximately 0.25 (20%/80%); very much the same as for the PANC1 cells *in vitro* (Fig 3C). The ratios in treatment group 5 (construct 132, 5 μg) ranged from 0.57 (36.14%/63.86%) to 1.9 (65.68%/34.32%); in the higher dose group (group 6; construct 132, 25 μg) the ratios were 3.6 (78.37%/21.63%), 5.8 (85.23%/14.77%) and no detectable mutant transcripts in sample 6A.

*KRAS*^mut^ basal signaling through the RAF/MEK/ERK pathway differentially regulates EGFR tyrosine and threonine autophosphorylation resulting in negative feedback control as demonstrated in a variety of *KRAS* mutated tumors [18–22]. Therefore, we examined the *in vivo* PANC1 mouse xenograft tumors (used for RFLP) to document the pattern of EGFR autophosphorylation (Western immunoblot). The total EGFR expression level in treated tumors averaged less than a 2-fold difference compared to untreated tumors (Fig 5B, panel c). On the other hand, in the construct 132 treated group there was a significant increase in the activating Y1068 EGFR phosphorylation site (Fig 5B, panel b) as well as in EGFR tyrosine phosphorylation sites Y1045, Y1068 and Y1125 (Fig 5C and S5A Fig). We also examined MEK, ERK and AKT expression and phosphorylation (S5B Fig). pMEK and pERK were somewhat higher in higher dose treated tumors (S5C Fig, panel a, treatment groups 4 and 6) but without change in pAKT at S473 and only slightly lowered at site T308 (S5D Fig). Protein levels for ERK and AKT were slightly lower (S5D Fig). Differing from most reported *in vitro* knockdown studies [7, 23–25], total RAS protein expression was about the same for treated versus untreated group (Fig 5B, panel a). The absence of non-target *KRAS*^*wt*^ knockdown minimizes the risk of toxicity.

## Discussion

The overall mutational frequency of *KRAS* in cancer is 22% but with non-uniform distribution amongst different cancer types. Greater than 90% of PDAC carry *KRAS* mutations (with a relatively high mutant allele specific imbalance) and the mutation frequency in lung and colorectal cancer are approximately 30–50% and 40–50%, respectively (COSMIC database). *KRAS* isoform specific mutation frequency likewise varies with cancer type, as do downstream signaling processes. This report demonstrates a novel purposefully designed *KRAS* multi-mutation genotype specific knockdown moiety with the potential for a systemically delivered therapeutic approach in a majority of patients with pancreas cancer. Previous studies with PC-7 (*KRAS*^G12V/G12V^) and PANC1 (*KRAS*^G12D/wt^) xenografts have shown that siRNA vectors targeting *KRAS* codon-12 mutations can be effective in reducing tumor growth when injected intratumorally [26]. Others have extended this approach to humans by combining an intratumoral siRNA vector with systemic chemotherapy to treat locally advanced, unresectable pancreatic cancers [27]. This was shown to be safe, well tolerated and with preliminary demonstration of clinical benefit; i.e. prolonged tumor control, shrinkage and biomarker reduction. However, given the high rate of early metastagenicity of pancreatic cancer and the advanced stage at diagnosis, local intratumoral injection of siRNA vectors is not a viable option for the majority of PDAC patients. AZD4785, an antisense oligonucleotide that indiscriminately targets *KRAS*^*mut*^ and *KRAS*^*wt*^ achieves effective mutant and wild-type knockdown both *in vitro* and *in vivo* by systemic delivery without demonstrable adverse effects [28]. Although the authors suggest that *NRAS* and/or *HRAS* compensate for *KRAS*^*wt*^ knockdown, it remains unclear whether or not the compartmentalization and functional specificity of the *RAS* isoforms will allow effective and safe clinical translation of this approach [29–31].

Both siRNA- and shRNA-mediated target gene expression knockdown have been shown to distinguish single nucleotide differences between alleles [32–34]. The position of the single nucleotide difference on the guide strand and the type of nucleotide matches are important factors determining efficacy. For siRNA, Schwarz et al. have shown that mismatches at guide strand positions 5, 9, 10, 12, 13 and 16 were most effective for differential expression knockdown, and that purine to purine mismatches are more discriminating than other mismatch types [32]. Specificity differences due to nucleotide positioning on the guide strand are possibly ascribable to target sequence composition, use of siRNA vs. shRNA, and/or different assay methods [32–35]. Rather than the seed region (proposed sequence recognition region), the central region, which contains the cleavage site for cleavage-dependent RNAi, was the most consistent site for sequence selectivity. Almost all *KRAS* mutant specific knockdown publications place the mutant sequence at the central region [23–25, 27, 36]. In addition to the central region, positions 4 and 16 of the guide strand were also found to be useful in sequence distinction [34, 35, 37]. Insofar as bi-shRNA-*KRAS*^*mut*^ utilizes both cleavage-dependent and cleavage-independent mechanisms, we decided to use the systematic tiling strategy to interrogate the positional effect that would result in the most effective, discriminatory, mutant-specific *KRAS* knockdown. Unlike most assay systems used by others that either attach the target sequence at the 3’ end of a reporter gene or attach the reporter sequence at the carboxyl terminus of a target gene or in polycistronic fashion, we inserted the *KRAS* coding sequence of the first 17 amino acids at the amino terminus of reporter genes to mimic the natural target gene sequence location with respect to both transcription and translation. Additionally, we placed both wild-type and mutant sequences on one dual luciferase reporter expression vector in order to compare WT and mutant knockdown in the same cell and environment. The insertion of the target sequence at the amino terminus did not affect the reporter gene expression. Positions 3 and 4 of the guide strand in the seed region rather than in the central region were the most discriminating. Notably, our *in vitro* growth inhibition study found placing the mutated nucleotide at the central region not only affected *KRAS*^*G12D*^ mutation cells (PANC1), but also *KRAS*^*wt*^ cells (HEK293). That there was no growth effect for *KRAS*^*wt*^ HT29 cells may due to inefficient *in vitro* transfection. The results from reporter assays were successfully translated to both *in vitro* and *in vivo* studies that showed effective knockdown of the mutant transcript without affecting the wild-type transcript in a native environment.

Onco-relevant RAS downstream signaling is complex and appears to be primarily mediated via three effector pathways: 1) Raf-MEK-ERK, 2) PI3k-AKT-mTOR, and 3) RalGEF-Ral with extensive pathway cross talk and regulatory feedback pathways [18–22]. Pathway utilization patterns have been shown to be mutation specific. For example, NSCLC cell lines with *KRAS*^*G12D*^ show activated PI3k and MEK whereas those with *KRAS*^*G12C*^ and *KRAS*^*G12V*^ show activated Ral and decreased AKT [38]. In addition, pathway utilization patterns are also tumor type context specific. MEK/ERK inhibition in lung adenocarcinoma lines with *KRAS*^*G12C*^ decreases EZH2 expression but is without affect in cell lines with *KRAS*^*G12V*^. On the other hand, MEK/ERK inhibition decreases EZH2 expression in colon and pancreatic cancer lines with both *KRAS*^*G12C*^ and *KRAS*^*G12V*^ [39]. There are also data indicating the potential therapeutic relevance of regulatory feedback systems within these pathways. As an example, constitutively active *KRAS*^*mut*^ regulates basal MEK signaling [7, 40], which in turn has a negative feedback effect on EGFR activity by enhancing inhibitory phosphorylation (e.g., T669) and relieving activating phosphorylation (e.g., Y1068, Y1069, and Y1125) at functionally specific binding sites [14, 24, 40]. Consequently, *KRAS*^*mut*^ downregulation facilitates activated EGFR-mediated RAS^wt^-GDP→RAS^wt^-GTP configuration as seen in mut/WT cell lines and increases NRAS-GTP levels in mut/WT and mut/- cell lines [14, 40]. In addition to being heteroallelic (*KRAS*^*G12D/wt*^) PANC1 cells exhibit higher EGFR copy number than other pancreatic cancer cell lines (PANC1 > MIA PaCa-2 > Capan-2). Data from our *in vivo* mutant-specific bi-shRNA treated PANC1 tumors show significantly increased p-EGFR with phosphorylation at Y1068 (Y1069), Y1045 and Y1125 sites, compared to vehicle treated or untreated tumors, without significant changes in total EGFR protein (clearly demonstrating enhanced tyrosine kinase activity due to post-translational modification induced activation rather than to increased protein expression). These data, as well as significant differential gene expression patterns (which will be presented in a separate paper) confirm that expression of the mutant *KRAS* allele was effectively and specifically suppressed. The consequent reactivation of EGFR signaling suggests a potential therapeutic benefit from combinatorial bi-shRNA^*KRAS mut*^ and EGFR inhibition. The RFLP data reveal that whereas the stable *KRAS*^*mut*^ mRNA was significantly repressed in treated tumor and PANC1 cells *in vitro* the *KRAS*^*wt*^ mRNA population is proportionally increased. These RFLP mRNA data are consistent with the lack of effect of our knockdown constructs on total *KRAS* protein, e.g., group 6 in Fig 5B, panel a, despite > 80% suppression of the mutant *KRAS* allele (Fig 5A). These findings differ from other *KRAS* mutant specific knockdown studies that show reduction of total KRAS protein [23–25] as well as reduction of both stable *KRAS*^*wt*^ and *KRAS*^*mut*^ mRNA [24] and, therefore, require further investigation. Insofar as the commonly used anti-RAS antibodies do not differentiate between RAS protein isoforms it may be that suppression of *KRAS*^*mut*^ expression relieves the negative feedback *KRAS*^mut^ →NRAS (and, possibly, both HRAS and NRAS) pathway[s] [40] thereby stabilizing total RAS expression. Other potentially contributory mechanisms to consider are 1) differences between studies in procedures and xenograft genotypes/phenotypes and their derivative signaling pathways [41], 2) the presence of extensive intratumoral heterogeneity as seen in recent CTC single cell expression data [42], 3) a lower *KRAS mut* ratio [43], 4) a *KRAS mut* effect on stem cell distribution (which would also account for epithelial-mesenchymal (EMT) shift) [44, 45], and 5) the stochastic, non-determinant loss of *KRAS mut* stem cells [44].

A major obstacle thus far preventing translation of RNAi technology to the clinic has been lack of effective systemic delivery comprising distribution, metabolism/elimination and tissue/intracellular entry. Using a non-targeted, fusogenic lipoplex [46, 47], we show that payload plasmid DNA is effectively delivered to tumors *in vivo* in functionally adequate concentrations. Although a freeze-dried formulation has the advantage of long-term stability allowing for storage and transport, heterogeneity and inconsistency in reconstituted material is problematic. In our experience the physical properties of the reconstituted material vary from batch to batch, often resulting in larger aggregates of test agents which require an additional filtration process to eliminate large particles (1, panel A). This makes it difficult to determine the concentration of the actual delivered final product. Additionally, batch-to-batch variation is difficult to control. In our initial tests of constructs 131 and 132, *in vitro* (PANC-1) studies showed both constructs capable of selective knockdown of the *KRAS* mutant allele expression without affecting wild-type allele whereas the *in vivo* study showed that construct 131 was not as effective as 132. Subsequent analysis of intratumoral DNA revealed that 131 was not as efficiently delivered as 132, supporting the inconsistency of the freeze-dried formulation. We have since developed a new lipoplex formulation process using the ethanol injection method and are in the process of completing optimization studies prior to GMP product manufacturing and large animal toxicology studies. The resulting product has a narrower volumetric range with a smaller average diameter, homogenous physical properties (Table 1, panel B), an efficient delivery and a process that is scalable.

**Table 1:** Physical properties of reconstituted material comparison.

**A. Freeze Dried DNA-LPX Batches**												
Batch #	LP Method	LP Buffer	DNA Payload	Product	Process Step	OD400	Z-Avg Size (d.nm)	PDI	Zeta (mV)	Int Mean (d.nm)	Vol Mean (d.nm)	Di90 (d.nm)
011514-A	Thin Film	10% Sucrose	pGBI-131	pGBI-131 DNA-LPX	Pre Freeze Dry	0.553	233.5	0.244	74.0	351.0	610.6	759
					Reconstituted	0.645	284.9	0.377	71.3	630.1	1033.0	720
					Extruded	0.611	257.8	0.258	65.6	477.6	746.5	613
011514-B			pGBI-132	pGBI-132 DNA-LPX	Pre Freeze Dry	0.667	262.5	0.343	72.8	483.5	749.9	754
					Reconstituted	0.690	295.3	0.425	69.9	628.0	1049.0	782
					Extruded	0.667	270.5	0.281	71.1	428.3	646.7	686
040914-A			pGBI-131	pGBI-131 DNA-LPX	Pre Freeze Dry	0.467	210.7	0.229	70.9	354.9	557.0	432
					Reconstituted	0.773	289.2	0.400	64.0	671.4	1098.0	938
					Extruded	0.635	263.5	0.281	60.9	427.9	731.6	918
040914-B			pGBI-132	pGBI-132 DNA-LPX	Pre Freeze Dry	0.457	212.6	0.172	70.4	261.7	263.9	439
					Reconstituted	0.899	323.3	0.553	63.7	751.7	1239.0	1720
					Extruded	0.723	289.9	0.274	63.5	509.5	795.6	735
**B. EtOH Injection DNA-LPX Batches**												
Batch #	LP Method	LP Buffer	DNA Payload	Product	Process Step	OD400	Z-Avg Size (d.nm)	PDI	Zeta (mV)	Int Mean (d.nm)	Vol Mean (d.nm)	Di90 (d.nm)
030315_	EtOH	5% Dextrose	pGBI-140	pGBI-140 DNA-LPX	Release	0.633	179.7	0.221	69.7	n/a	n/a	375
030415_					Release	0.656	184.2	0.262	68.6	n/a	n/a	435
030915_					Release	0.498	165.8	0.218	71.6	n/a	n/a	363
031815B-P					Intermediate	0.417	146.2	0.233	72.0	282.1	358.5	319
					Release	0.427	148.3	0.203	69.2	188.6	136.6	321
050516_					Intermediate	0.444	154.7	0.225	62.7	218.2	221.6	330
					Release	0.386	152.6	0.228	61.3	233.9	235.9	351
022317_					Intermediate	0.380	145.2	0.212	60.6	184.0	126.0	313
					Release	0.443	148.2	0.205	59.2	225.2	222.9	313

The modular, multi mutant-specific bi-shRNA^*KRAS*^ herein described represents a unique therapeutic approach to cancer, including those with multiple mutant heteroalleles and/or those with two or more synthetic lethals. The safety and biodistributiion of the systemically delivered fusogenic lipoplex is currently being evaluated in a phase I clinical trial of bi-shRNA^*EWS/FLI1*^ in patients with Ewing’s sarcoma (BB-IND 16939). Insofar as the lack of *KRAS*^*mut*^ druggable sites, the multifarity of downstream signaling pathways and the lack of a safe, efficient, and systemic tumor selective delivery vehicle have stymied the development of a translatable targeted treatment for *KRAS* mutated cancers, the bi-shRNA^*KRAS*^ lipoplex, by addressing these obstacles, is primed for clinical implementation.

## Supporting information

S1 Fig Schematics illustrate RFLP method to determine proportion of mutant and wild-type *KRAS* transcripts. Schematics shows PCR primers used to amplify the target mRNA sequence region. PCR primers generated Bst XI recognition sequence for mRNA with wild-type allele sequence, but not for the mutant allele sequence.
(TIFF)

S2 Fig psiCHECK2 based test reporter vector sequence arrangement and demonstration of the test reporter vector. **A.** Sequence inserted into the psiCHECK2 vector and designated code for each test reporter vector. Sequence in green is coding sequence for amino acids G12 and G13.
**B.** Bar graph show RL/FF RLU ratio when co-transfection of test reporter vector and knockdown vectors at different molar ratio; test vector to knockdown vector ratio at (A) 4:1, (B) 1:1, (C) 2:1, or (D) 1:2. Sample 1 is the control sample without knockdown vector. Samples 2–7 are 6 different knockdown constructs. Red bar show average control sample ratio. Standard deviation bar represents measurement from quadruplet samples of independently transfected cells in 96-well format and assayed simultaneously post-transfection.
**C.** Bar graph show % knockdown of wt vs. mu for placing mutant sequence at different position of the guide strand. P2 = position 2, P3 = position 3 and so forth. % knockdown is determined against RLU of control sample transfected with test reporter vector only. Standard deviation bar represents measurement from quadruplet samples of independently transfected cells in 96-well format and assayed simultaneously post-transfection.
(TIF)

S3 Fig Expression unit sequences of triple knockdown constructs and the schematics of establishing stable transformant of PANC-1 cells with triple knockdown constructs. **A.** Expression unit sequence for triple knockdown constructs in miR30a backbone with miR17-92 gap sequence. Pink letters are passenger strand sequence. Green letters are guide strand sequence.
**B.** Expression unit sequence for triple knockdown constructs in miR17-92 backbone. Pink letters are passenger strand sequence. Green letters are guide strand sequence.
**C.** Schematics show co-transfection process to generate PANC-1 cell transformant clones transformed with triple knockdown vectors.
(TIF)

S4 Fig Compare cell growth effect by *KRAS* mutant specific knockdown vectors. **A.** HEK-293 cells were transfected with various constructs at 10 ng of plasmid DNA per well in 96-wells plates at 8 replicates per sample. Lane 1 = Control transfected by pUMVC3 empty vector. Lanes 2–11 were transfected by constructs with G12D mutation at position 2–11 of the guide strand. Lane 12 is no transfection control. Cells were lysed at 24 hrs post-transfection and assayed by CellTiter Blue kits. X-axis is sample numbers. Y-axis is OD 570 units. Standard deviation bar represents measurements tumors from 8 replicates. Two-tailed equal variances student T-test is used for ρ-value evaluation.
**B.** PANC-1 cells were transfected with various constructs at 10 ng of plasmid DNA per well in 96-well plates at 8 replicates per sample. Lane 1 = Control transfected by pUMVC3 empty vector. Lanes 2–11 were transfected by constructs with G12D mutation at position 2–11 of the guide strand. Lane 12 is no transfection control. Cells were lysed at 24 hrs post-transfection and assayed by CellTiter Blue kits. X-axis is sample numbers. Y-axis is OD 570 units. Standard deviation bar represents measurements tumors from 8 replicates. Two-tailed equal variances student T-test is used for ρ-value evaluation.
(TIF)

S5 Fig Western immunoblots of *in vivo* tumor samples. **A.** Western immunoblot summarize p-EGFR and EGFR expressions in *in vivo* tumor samples examined. Treatment grouping and sample grouping is the same as shown in Fig 4
**B.** Western immunoblot summarize p-EGFR and EGFR expressions in *in vivo* tumor samples examined. Treatment grouping and sample grouping is the same as shown in Fig 4
**C.** Bar graphs show relative fold levels of p-Mek (panel a), Mek (panel b), p-Erk (panel c), and Erk (panel d) for treatment groups 1–6. Phosphorylation level was normalized with total Mek or Erk protein. Total Mek or Erk protein level was normalized with GAPDH. Treatment grouping is the same as shown in Fig 4. Standard deviation bar represents measurements tumors from three animals. One-tailed equal variances student T-test is used for ρ-value evaluation.
**D.** Bar graphs show relative fold levels of p-Akt at amino acid T308 (panel a), p-Akt at amino acid S473 (panel b), and Akt (panel c) for treatment groups 1–6. Phosphorylation level was normalized with total Akt protein. Total Akt protein level was normalized with GAPDH. Treatment grouping is the same as shown in Fig 4. Standard deviation bar represents measurements from three animals.
(TIF)

S1 Table Nucleotide screening position. Summary table shows the tiling position of each single target knockdown vectors and their knockdown efficiency in respect to wild-type (Wt) and mutant (Mu) sequences. Red highlights show the position of single nucleotide matching mutant sequence within the guide strand sequence (mis-match to wild type sequence). Blue and purple highlights show the position of an additional mismatched sequences in the guide strand of the knockdown vector for potential knockdown enhancement. Yellow highlights show the constructs with widest difference in mutant and wild-type knockdown efficiency.
(DOCX)
