# Supplementary figures and images for: KRAS mutant allele-specific expression knockdown in pancreatic cancer model with systemically delivered bi-shRNA KRAS lipoplex
Source: PLoS One. 2018 May 31;13(5):e0193644. doi: 10.1371/journal.pone.0193644 (PMC5979018; doi:10.1371/journal.pone.0193644)

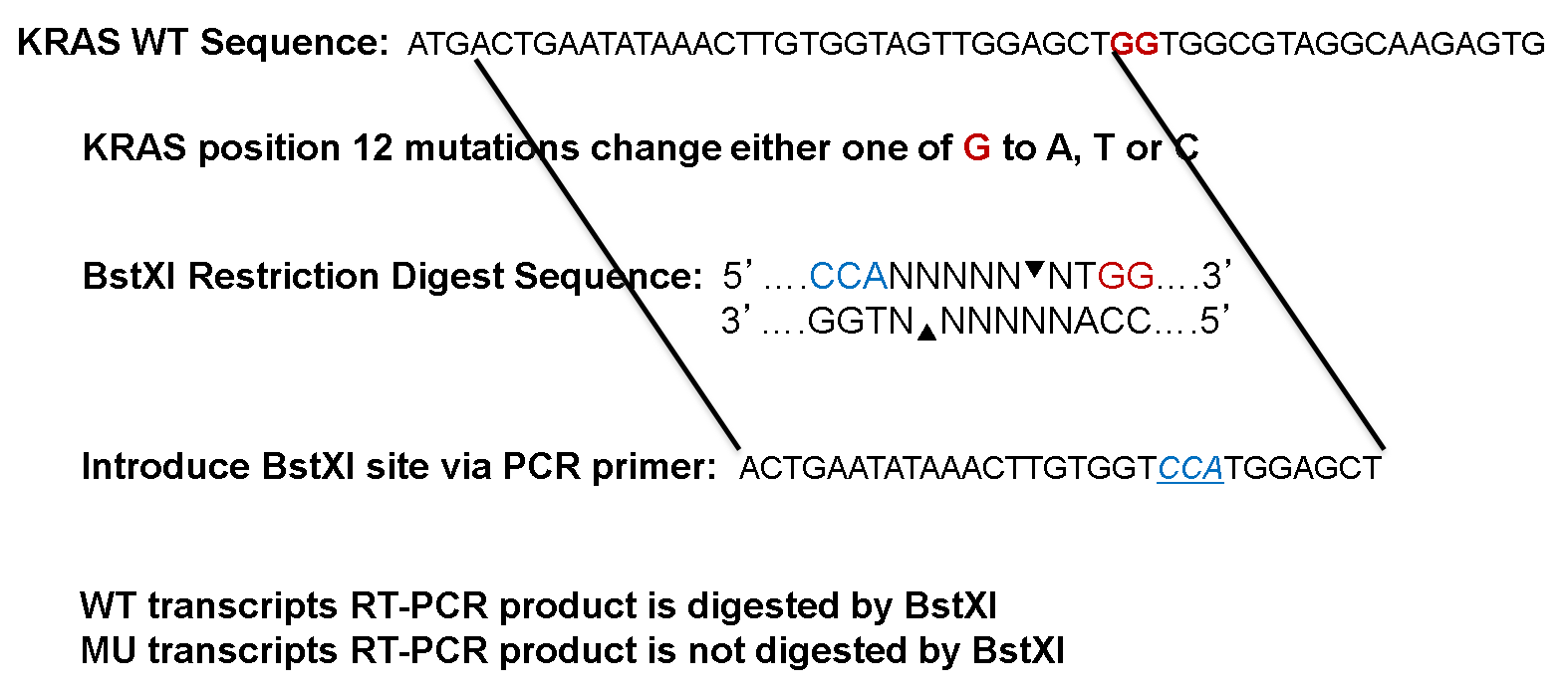

Supplement: S1 Fig — Schematics shows PCR primers used to amplify the target mRNA sequence region. PCR primers generated Bst XI recognition sequence for mRNA with wild-type allele sequence, but not for the mutant allele sequence. (TIFF) [file pone.0193644.s001.tiff]

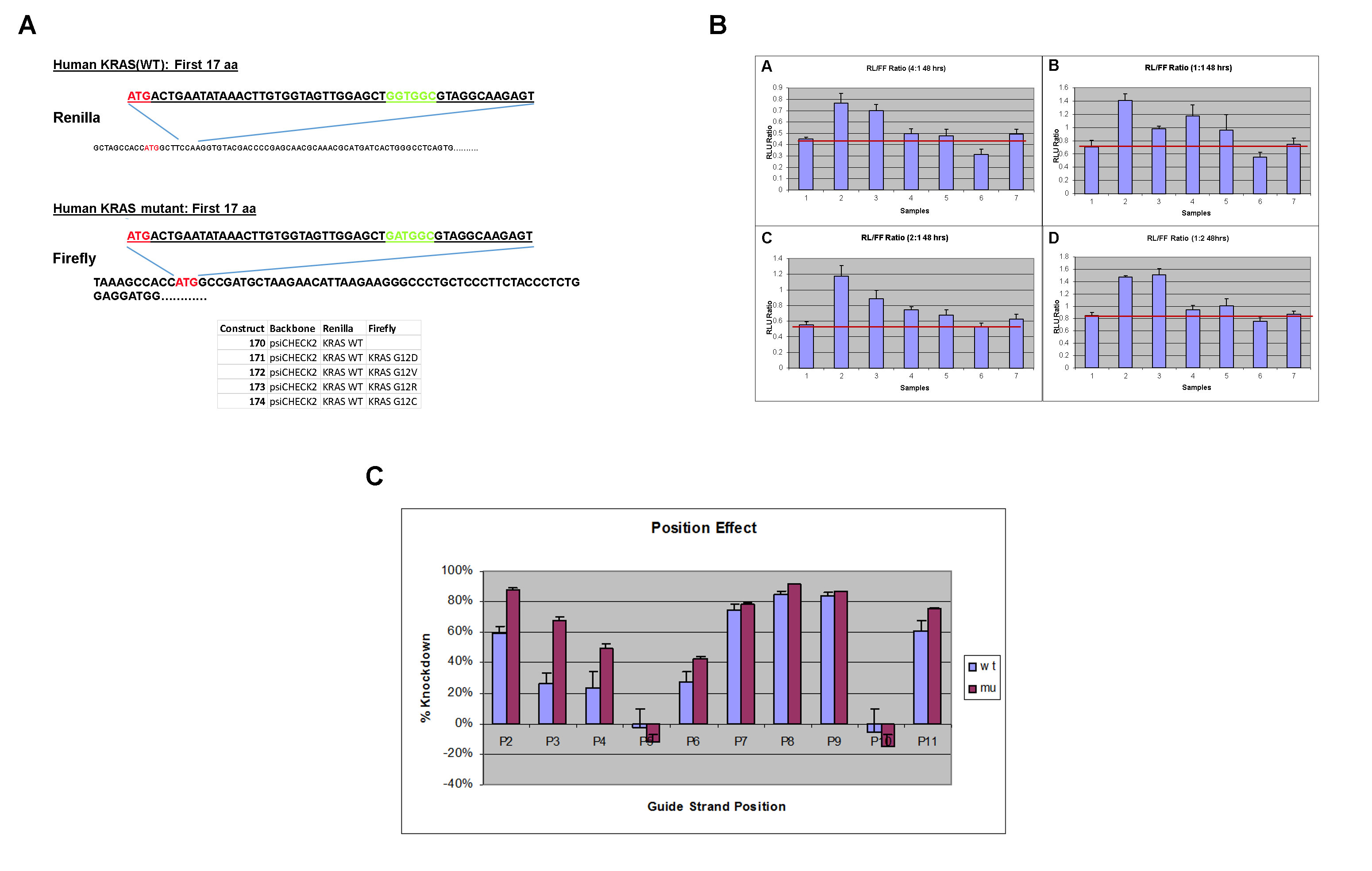

Supplement: S2 Fig — A. Sequence inserted into the psiCHECK2 vector and designated code for each test reporter vector. Sequence in green is coding sequence for amino acids G12 and G13. B. Bar graph show RL/FF RLU ratio when co-transfection of test reporter vector and knockdown vectors at different molar ratio; test vector to knockdown vector ratio at (A) 4:1, (B) 1:1, (C) 2:1, or (D) 1:2. Sample 1 is the control sample without knockdown vector. Samples 2–7 are 6 different knockdown constructs. Red bar show average control sample ratio. Standard deviation bar represents measurement from quadruplet samples of independently transfected cells in 96-well format and assayed simultaneously post-transfection. C. Bar graph show % knockdown of wt vs. mu for placing mutant sequence at different position of the guide strand. P2 = position 2, P3 = position 3 and so forth. % knockdown is determined against RLU of control sample transfected with test reporter vector only. Standard deviation bar represents measurement from quadruplet samples of independently transfected cells in 96-well format and assayed simultaneously post-transfection. (TIF) [file pone.0193644.s002.tif]

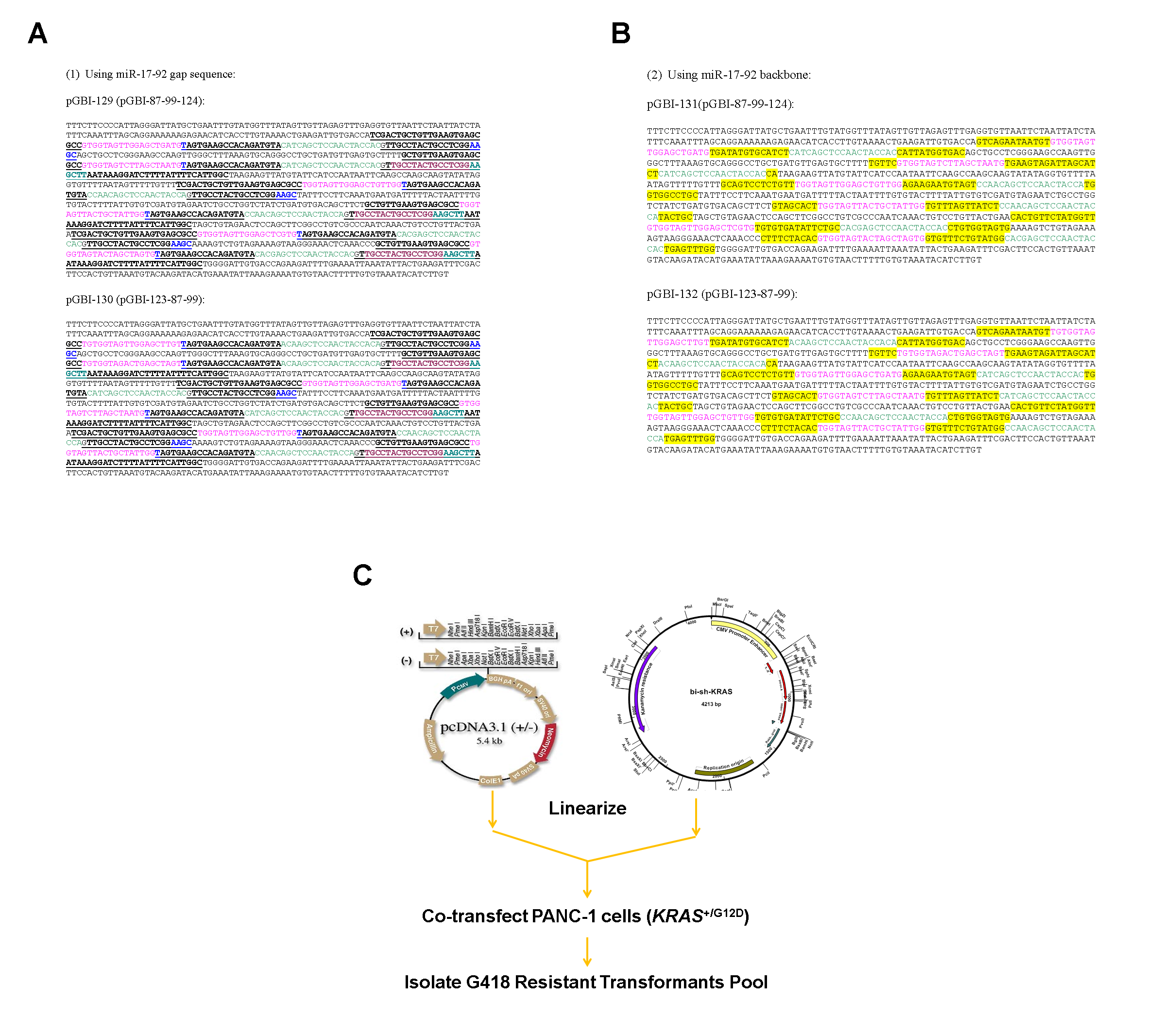

Supplement: S3 Fig — A. Expression unit sequence for triple knockdown constructs in miR30a backbone with miR17-92 gap sequence. Pink letters are passenger strand sequence. Green letters are guide strand sequence. B. Expression unit sequence for triple knockdown constructs in miR17-92 backbone. Pink letters are passenger strand sequence. Green letters are guide strand sequence. C. Schematics show co-transfection process to generate PANC-1 cell transformant clones transformed with triple knockdown vectors. (TIF) [file pone.0193644.s003.tif]

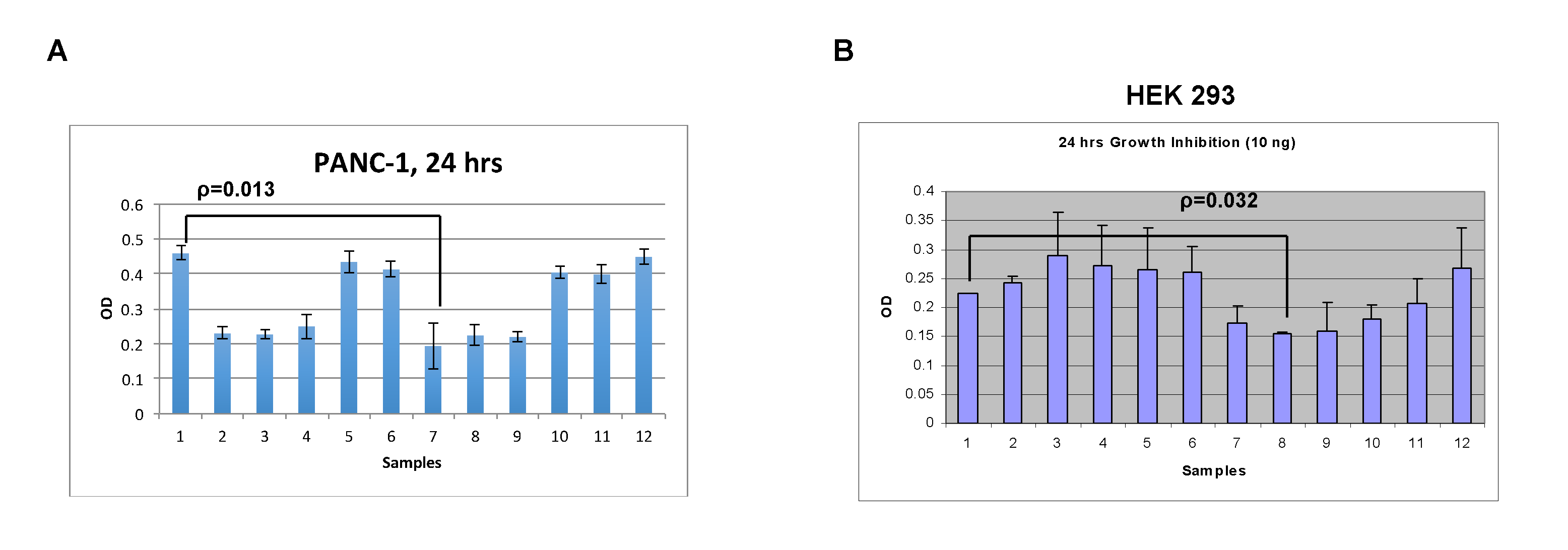

Supplement: S4 Fig — A. HEK-293 cells were transfected with various constructs at 10 ng of plasmid DNA per well in 96-wells plates at 8 replicates per sample. Lane 1 = Control transfected by pUMVC3 empty vector. Lanes 2–11 were transfected by constructs with G12D mutation at position 2–11 of the guide strand. Lane 12 is no transfection control. Cells were lysed at 24 hrs post-transfection and assayed by CellTiter Blue kits. X-axis is sample numbers. Y-axis is OD 570 units. Standard deviation bar represents measurements tumors from 8 replicates. Two-tailed equal variances student T-test is used for ρ-value evaluation. B. PANC-1 cells were transfected with various constructs at 10 ng of plasmid DNA per well in 96-well plates at 8 replicates per sample. Lane 1 = Control transfected by pUMVC3 empty vector. Lanes 2–11 were transfected by constructs with G12D mutation at position 2–11 of the guide strand. Lane 12 is no transfection control. Cells were lysed at 24 hrs post-transfection and assayed by CellTiter Blue kits. X-axis is sample numbers. Y-axis is OD 570 units. Standard deviation bar represents measurements tumors from 8 replicates. Two-tailed equal variances student T-test is used for ρ-value evaluation. (TIF) [file pone.0193644.s004.tif]

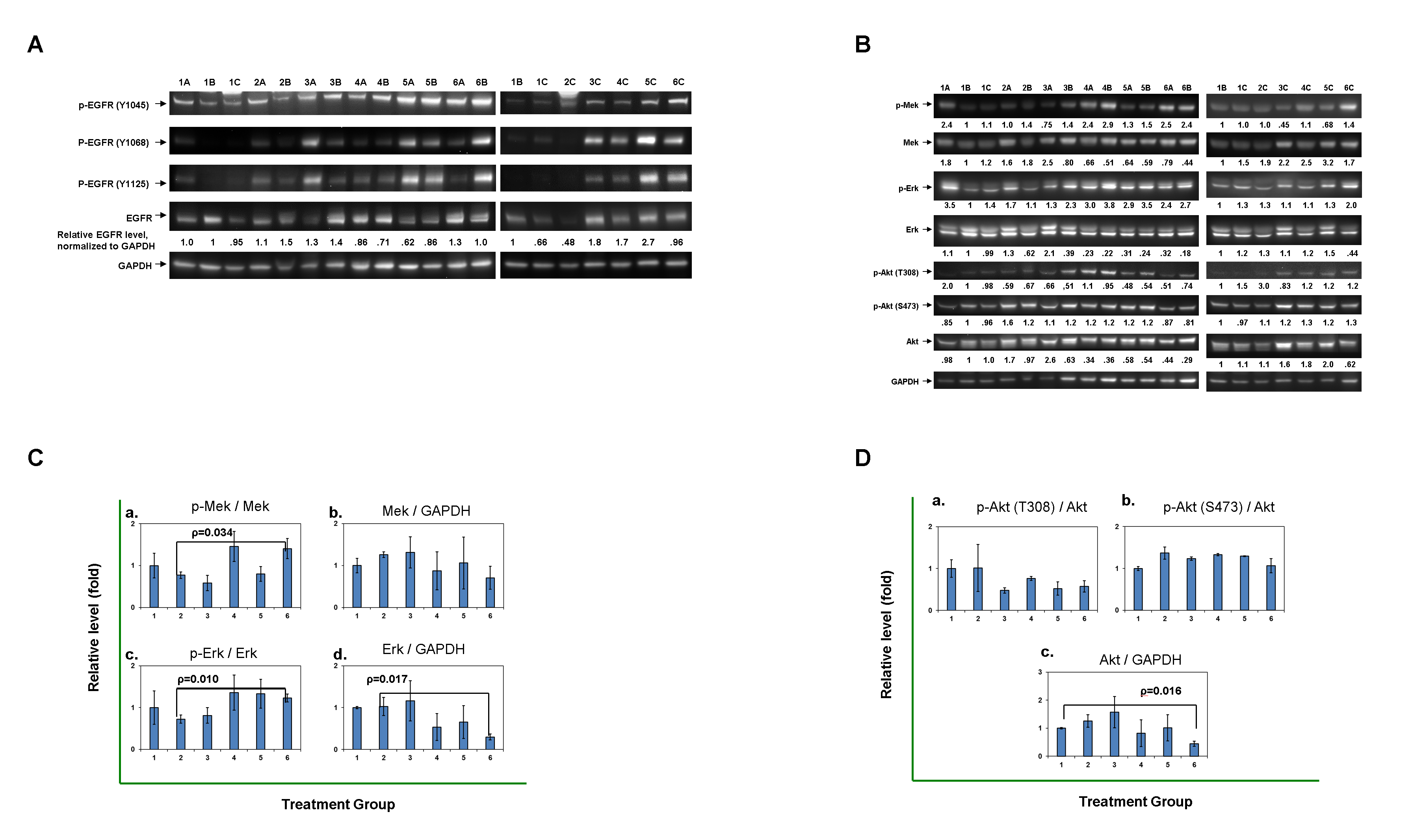

Supplement: S5 Fig — A. Western immunoblot summarize p-EGFR and EGFR expressions in in vivo tumor samples examined. Treatment grouping and sample grouping is the same as shown in Fig 4 B. Western immunoblot summarize p-EGFR and EGFR expressions in in vivo tumor samples examined. Treatment grouping and sample grouping is the same as shown in Fig 4 C. Bar graphs show relative fold levels of p-Mek (panel a), Mek (panel b), p-Erk (panel c), and Erk (panel d) for treatment groups 1–6. Phosphorylation level was normalized with total Mek or Erk protein. Total Mek or Erk protein level was normalized with GAPDH. Treatment grouping is the same as shown in Fig 4. Standard deviation bar represents measurements tumors from three animals. One-tailed equal variances student T-test is used for ρ-value evaluation. D. Bar graphs show relative fold levels of p-Akt at amino acid T308 (panel a), p-Akt at amino acid S473 (panel b), and Akt (panel c) for treatment groups 1–6. Phosphorylation level was normalized with total Akt protein. Total Akt protein level was normalized with GAPDH. Treatment grouping is the same as shown in Fig 4. Standard deviation bar represents measurements from three animals. (TIF) [file pone.0193644.s005.tif]
