# Supplementary material for: KRAS mutant allele-specific expression knockdown in pancreatic cancer model with systemically delivered bi-shRNA KRAS lipoplex
Source: PLoS One. 2018 May 31;13(5):e0193644. doi: 10.1371/journal.pone.0193644 (PMC5979018; doi:10.1371/journal.pone.0193644)
Supplement: S1 Table — Summary table shows the tiling position of each single target knockdown vectors and their knockdown efficiency in respect to wild-type (Wt) and mutant (Mu) sequences. Red highlights show the position of single nucleotide matching mutant sequence within the guide strand sequence (mis-match to wild type sequence). Blue and purple highlights show the position of an additional mismatched sequences in the guide strand of the knockdown vector for potential knockdown enhancement. Yellow highlights show the constructs with widest difference in mutant and wild-type knockdown efficiency. (DOCX) [file pone.0193644.s006.docx]

**S1 Table. Nucleotide Screening Position.** Summary table shows the tiling position of each single target knockdown vectors and their knockdown efficiency in respect to wild-type (Wt) and mutant (Mu) sequences. Red highlights show the position of single nucleotide matching mutant sequence within the guide strand sequence (mis-match to wild type sequence). Blue and purple highlights show the position of an additional mismatched sequences in the guide strand of the knockdown vector for potential knockdown enhancement. Yellow highlights show the constructs with widest difference in mutant and wild-type knockdown efficiency.
